# Supplementary material for: Transcriptomic Analyses of Brains of RBM8A Conditional Knockout Mice at Different Developmental Stages Reveal Conserved Signaling Pathways Contributing to Neurodevelopmental Diseases
Source: Int J Mol Sci. 2023 Feb 27;24(5):4600. doi: 10.3390/ijms24054600 (PMC10003467; doi:10.3390/ijms24054600)
Supplement: Supplementary file 1 [file ijms-24-04600-s001.zip › ijms-2166106-supplementary.pdf]

Transcriptomic Analyses of Brains of RBM8A Conditional Knockout Mice at Different Developmental Stages Reveal Conserved Signaling Pathways Contributing to Neurodevelopmental Diseases

Colleen McSweeney [1,†], Miranda Chen [1], Fengping Dong [1,2,†], Aswathy Sebastian [3], Derrick James Reynolds [4], Jennifer Mott [1], Zifei Pei [1], Jizhong Zou [5], Yongsheng Shi [4] and Yingwei Mao [1,*]

Immunohistochemistry

Postnatal brains were collected on postnatal day 17 of the cKO mice. Mice were anesthetized with avertin (2.5% avertin), and perfused with artificial cerebrospinal fluid (119 mM NaCl, 26.2 mM $NaHCO_3$, 2.5 mM KCl, 1 mM NaH_2PO_4, 1.3 mM $MgCl_2$, 10 mM glucose) until the liver was clear of blood. Then the mice were briefly perfused with 4% paraformalydehyde (PFA). The brain was then dissected out, and post-fixed in 4% PFA for a minimum of 24 h at 4 °C. The brains were then sliced on the vibratome in 70 μm thick slices. Slices were stored in 6 well plates filled with PBS and sodium azide in 4 °C. For immunohistochemistry, slices were rinsed 3 times in PBS for 5 min while gently rocked at room temperature (RT). Then slices were blocked and permeabilized using 5% donkey serum in 0.3% PBST (1× PBST 8 g NaCl, 0.2 g KCl, 1.44 g NaH_2PO_4, 0.24 g KH_2PO_4, 3 mL Tween-20, fill to 1 L). During the blocking step, slices were gently rocked at RT. Next, slices were incubated in primary antibody Foxp2 (SCBT sc-21069, Dallas, TX, USA) diluted in 5% donkey serum in 0.3% PBST. Slices were shaken gently at RT over night.. The next day, slices were washed 3 times in PBS, then incubated for 1hr with the secondary antibody of the appropriate species, conjugated with Alexa Flour 488. Samples were rocked gently at RT and covered with foil to protect them from the light. Slices were then washed with PBS two more times for 5 min. Slices were then plated on mount slides with ProLong™ Gold anti-fade (Invitrogen, P36931. Waltham, MA, USA). Fluorescent images were captured using a Zeiss (White Plains, NY, USA) LSM 5 confocal microscope.

Figure S1

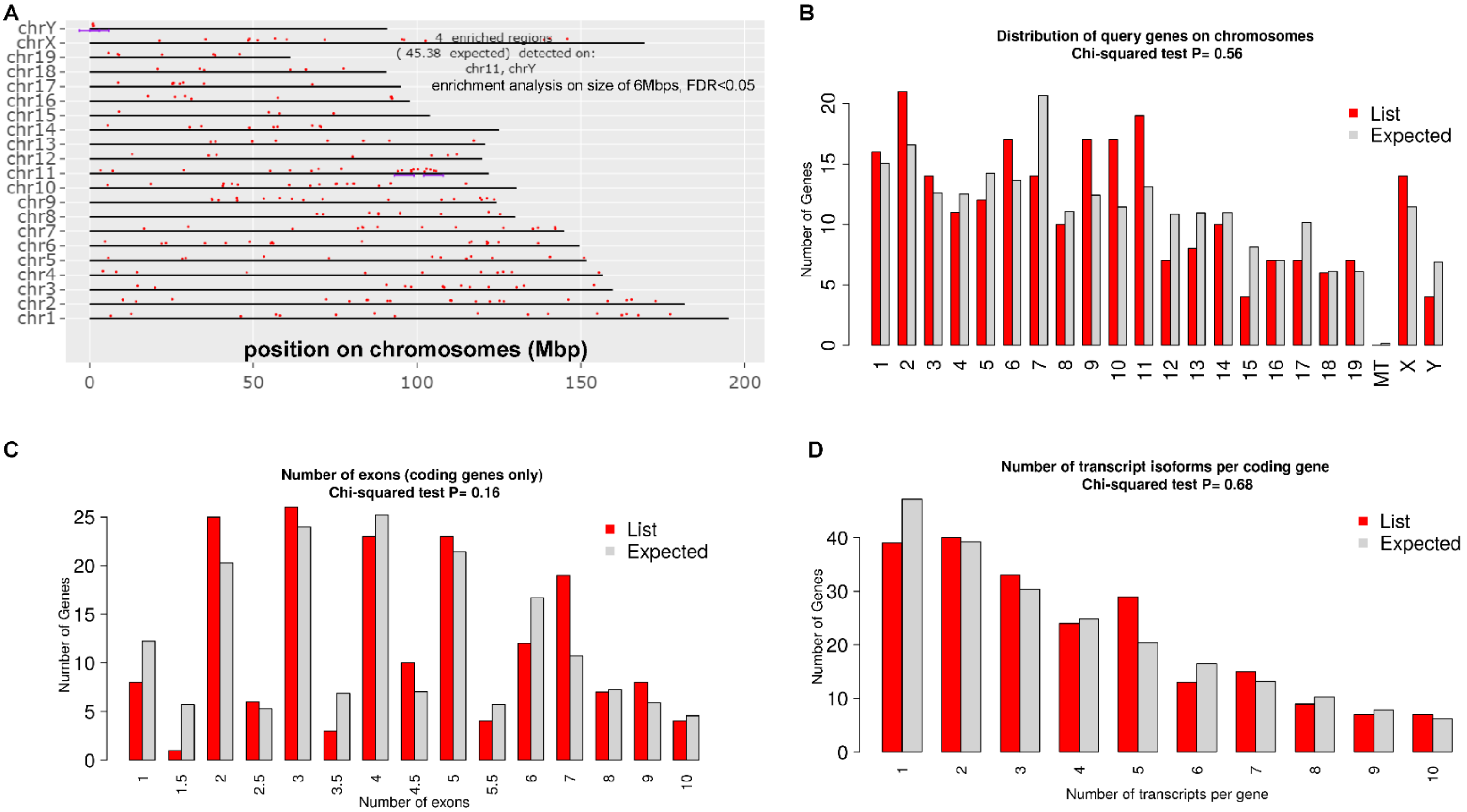

Figure S1. Analyses of overall DEG features from P17 RNAseq dataset. (A) Positions of DEGs in the different chromosomes. Four regions (marked with purple line) in chromosome 11 and Y show high density of DEG distributions (FDR<0.05). (B) DEGs from P17 dataset are evenly distributed on each chromosome. (C) Numbers of exons of DEGs from P17 dataset are not different from what is expected in the mouse genome. (D) Numbers of transcript isoforms per gene from P17 DEGs are not different from what is expected in the mouse genome.

Figure S2

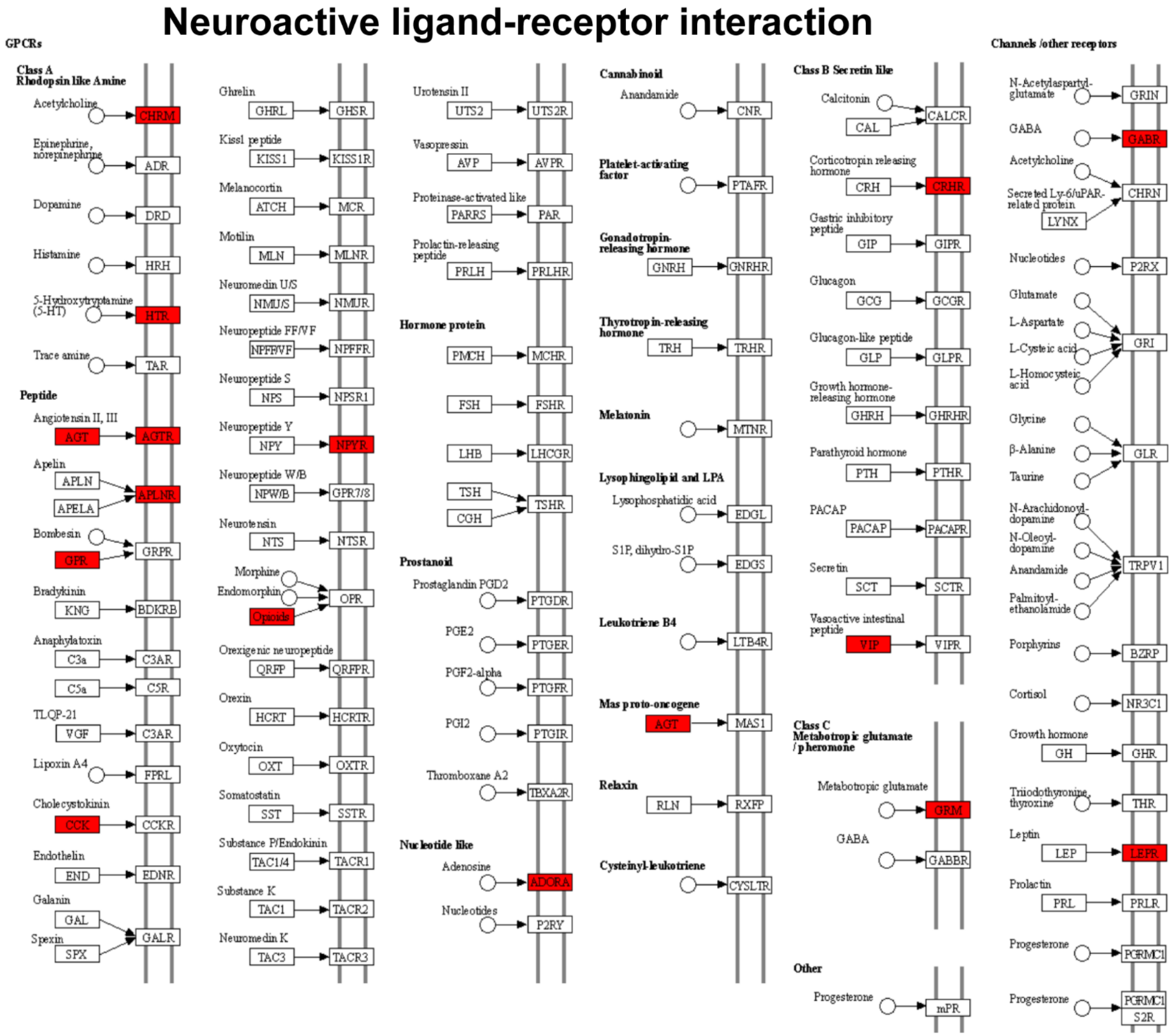

Figure S2. The diagram shows a significant KEGG pathway-Neuroactive ligand-receptor interaction with DEGs highlighted in red.

Figure S3

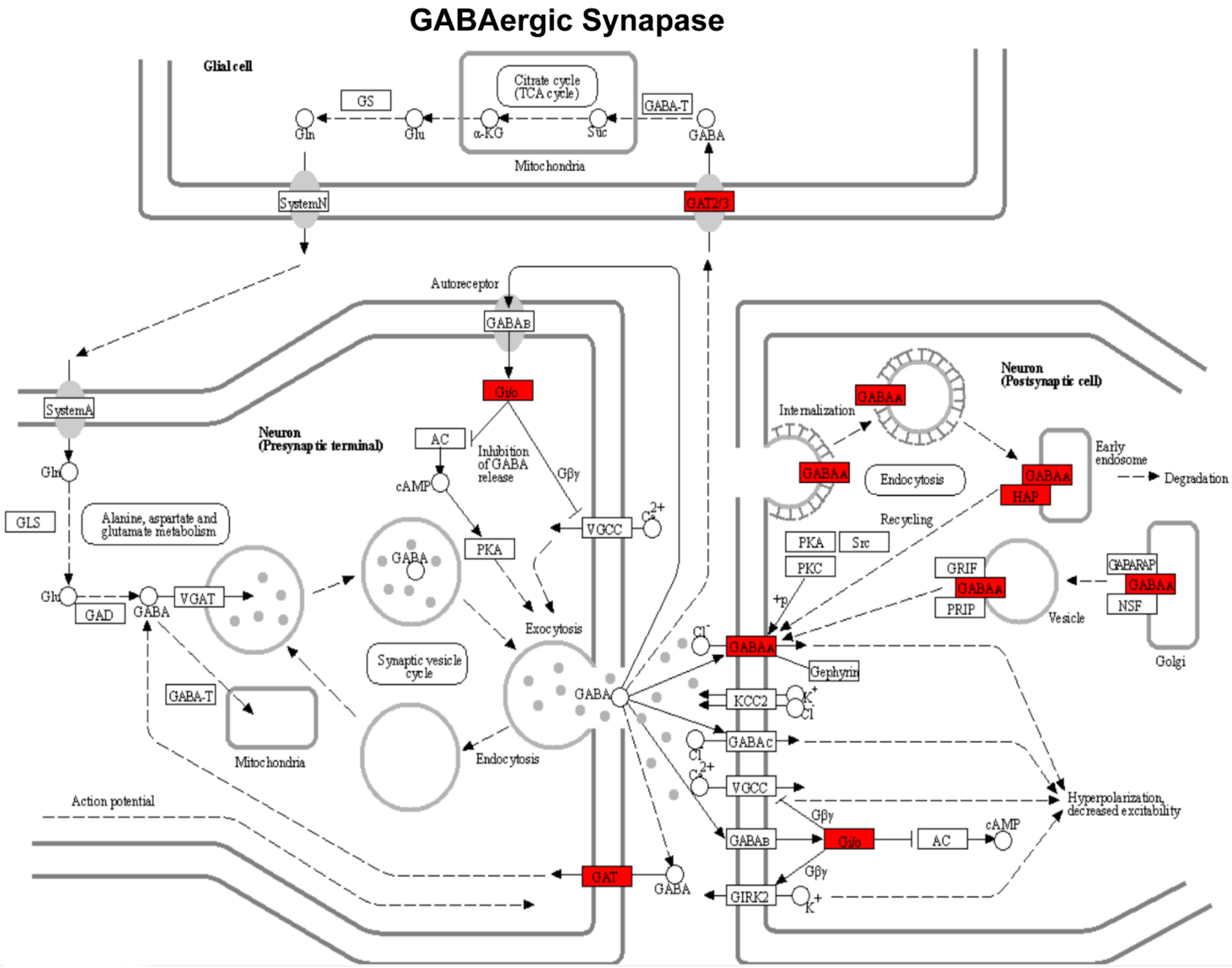

Figure S3. The diagram shows a significant KEGG pathway- GABAergic synapase with DEGs highlighted in red.

Figure S4

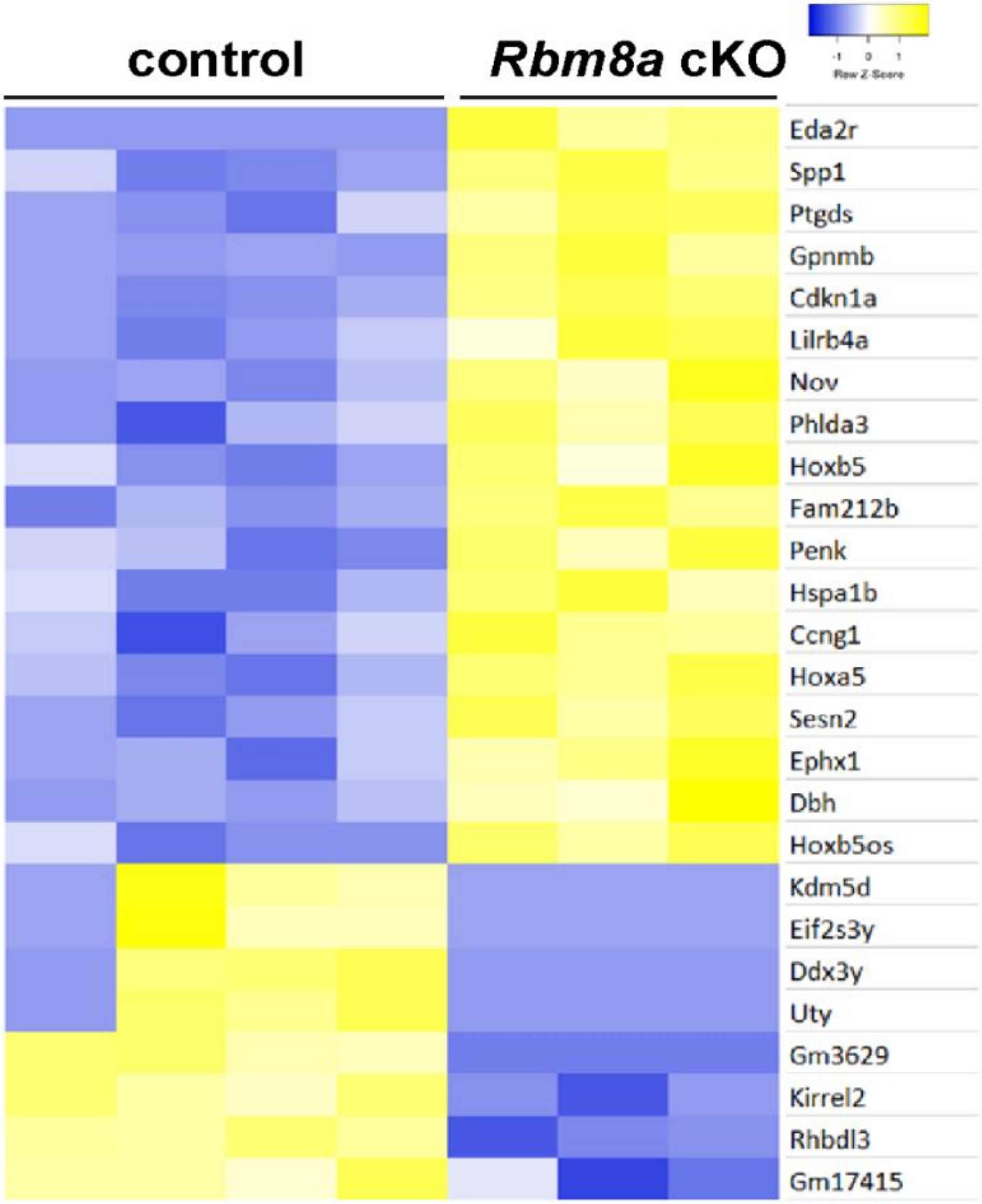

Figure S4: Heat map of RNA transcript readings for all genes with significant expressional changes in the E12 cKO mouse hindbrain (*q* < 0.05).

From the RNA-seq data, the transcript counts of each gene were compared among the six mice.

Figure S5

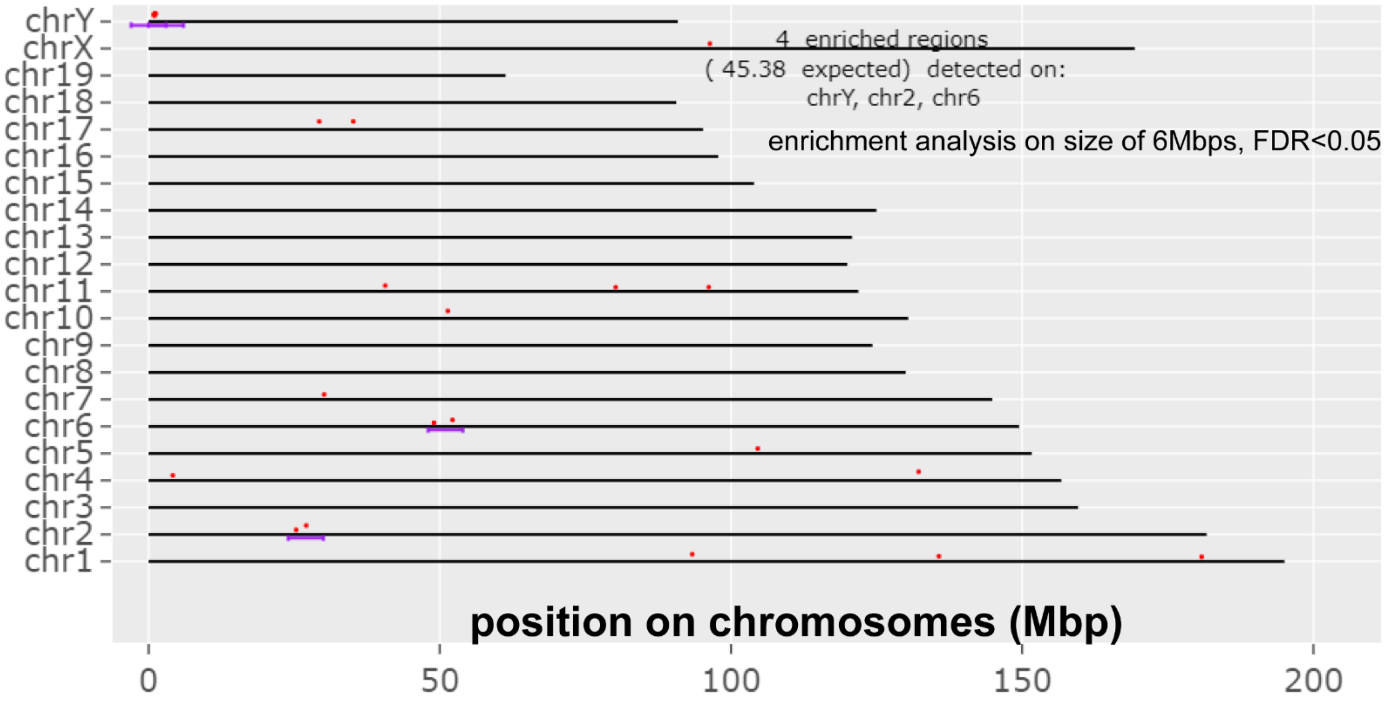

Figure S5: Positions of DEGs at E12 hindbrain in the different chromosomes. Four regions (marked with purple line) in chromosome 2, 6 and Y show high density of DEG distributions (FDR<0.05).

Figure S6

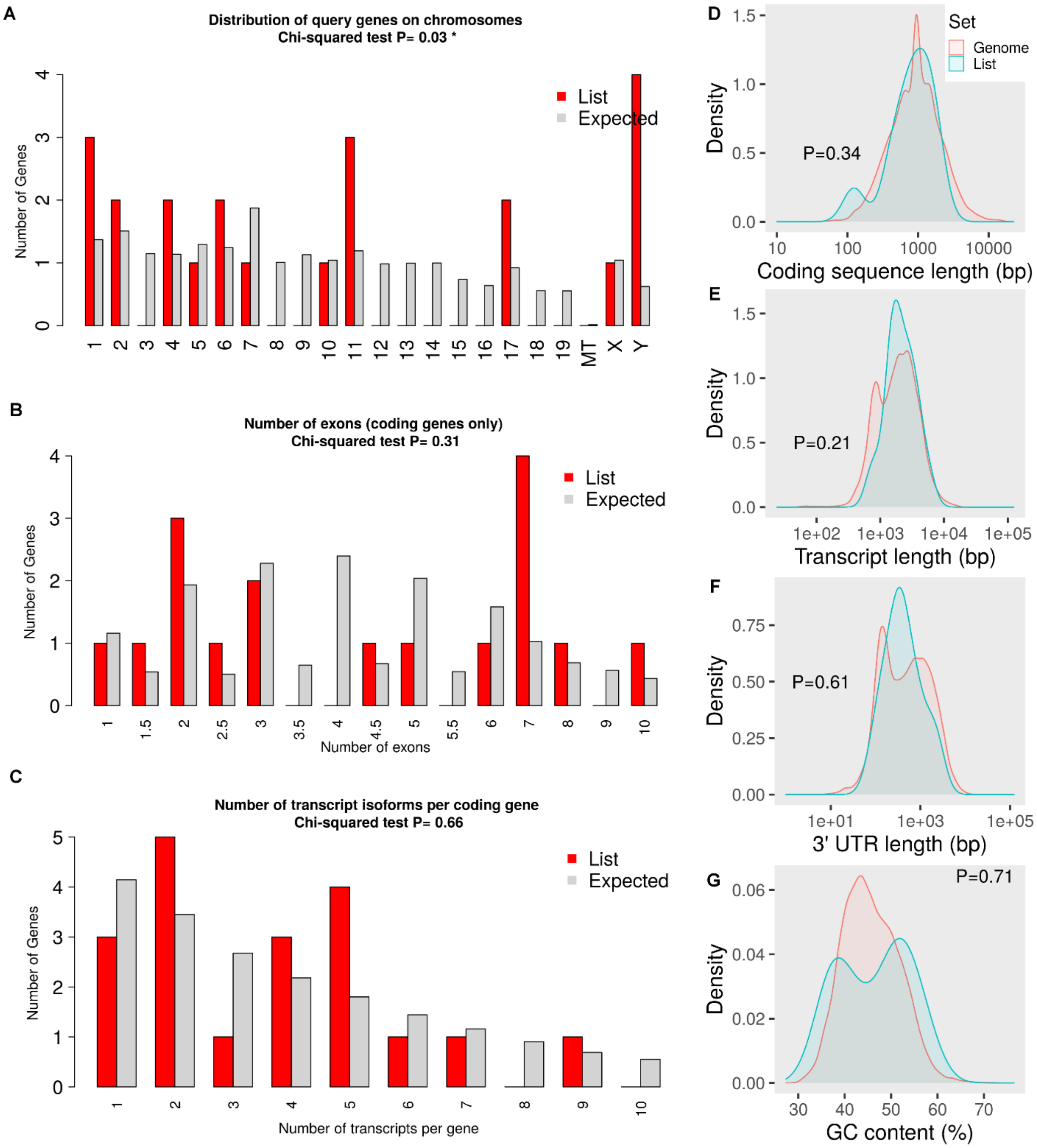

Figure S6. Analyses of overall DEG features from E12 hindbrain RNAseq dataset. . (A) DEGs from E12 hindbrain dataset are unevenly distributed on each chromosome. P=0.03. (B) Numbers of exons of DEGs from P17 dataset are not different from what is expected in the mouse genome. (C) Numbers of transcript isoforms per gene from P17 DEGs are not different from what is expected in the mouse genome. (D-G) DEGs at E12 hindbrain show no significant changes in coding sequence length (D), transcript length (E), 3′ UTR length (F), and GC content (G) when compared with general genome.

Figure S7

P53 signaling pathway

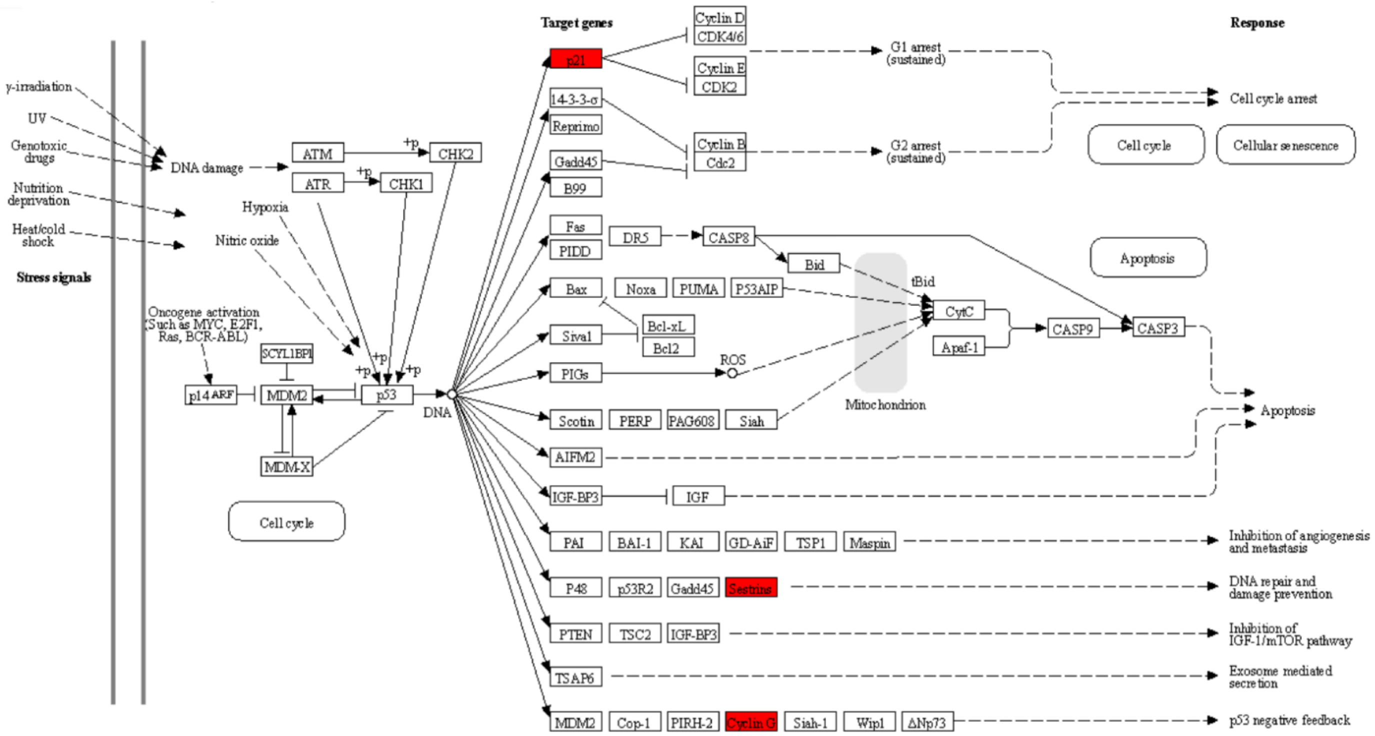

Figure S7. The diagram shows a significant KEGG pathway- P53 signaling pathway with DEGs highlighted in red for E12 hindbrain.

Table S1: Common upregulated DEGs between the cortex and hindbrain regions at E12.
The 10 genes that were upregulated in both the cortex and hindbrain are described briefly. Highlighted rows represent genes that are known to influence cellular proliferation.

Gene ID	Gene Name	Pathway and function
Fam212b (Ink2a)	Inka box actin regulator 2	Associated with rapidly-proliferating NPCs in the embryonic brain and with some immature neurons of the cerebral cortex, hippocampus, and cerebellum in the postnatal brain .[23]
Cdkn1a (p21)	Cyclin-dependent kinase inhibitor 1A	Binds a wide range of Cdks to negatively regulate cell cycle progression. Overexpression of Cdkn1a causes decreased cell proliferation. [34]
Ccng1	Cyclin G1	Participates in G2/M cell cycle arrest, damage recovery, and resumption of cell division. [37]
Ephx1 (mEH)	Microsomal epoxide hydrolase 1	Detoxifies xenobiotic compounds throughout the body; may also hydrolyze certain endogenous fatty acid epoxides. [39]
Sesn2	Sestrin 2	Protects cells from death during stress, especially during glucose starvation. Predicted to regulate mitochondrial metabolic processes. [37]
Phlda3	Pleckstrin homology like domain A3	Competitively binds the PH domain of Akt to negatively regulate cell proliferation. May also interact positively with p53. [36]
Eda2r	Ectodysplasin A2 receptor	Activated downstream of p53; may contribute to cell death pathways. [38]
Lilrb4a	Leukocyte immunoglobulin-like receptor B4A	Inhibitory receptor that suppresses LPS-mediated and T_H2-dependent inflammation in the immune response. [40]
Spp1	Secreted phosphoprotein 1 (Osteopontin)	An anti-inflammatory cytokine that participates in tissue remodeling, bone homeostasis, and wound repair.[41]
Gpnmb	Glycoprotein nmb (Osteoactivin)	Negative regulator of osteoclast differentiation. May speed up cancer progression when overexpressed.[42]
